# Polyphenol Composition, Antioxidant Capacity and Xanthine Oxidase Inhibition Mechanism of Furong Plum Fruits at Different Maturity Stages

**DOI:** 10.3390/foods12234253

**Published:** 2023-11-24

**Authors:** Zhipeng Zheng, Li Wu, Wei Deng, Kexin Yi, Yibin Li

**Affiliations:** 1 Institute of Food Science and Technology, Fujian Academy of Agricultural Sciences, Fuzhou 350003, China; 2 College of Food Science, Fujian Agriculture and Forestry University, Fuzhou 350002, China; 3 Key Laboratory of Subtropical Characteristic Fruits, Vegetables and Edible Fungi Processing (Co-Construction by Ministry and Province), Ministry of Agriculture and Rural Affairs, Fuzhou 350003, China; 4 Fujian Province Key Laboratory of Agricultural Products (Food) Processing Technology, Fuzhou 350003, China

**Keywords:** Furong plum, polyphenols, antioxidant, xanthine oxidase, inhibition kinetics

## Abstract

An experiment was conducted on the polyphenol content, flavonoid content, anthocyanin content, and antioxidant capacity of Furong plum (*Prunus salicina* Lindl. cv. “furong”) at different maturity stages to determine the most suitable maturity stage. The inhibition of plum polyphenols on xanthine oxidase (XOD) was measured, and its kinetics were studied to reveal the inhibitory mechanism. The experimental results showed that the polyphenol, flavonoid and anthocyanin contents of plums at the ripe stage were the highest, reaching 320.46 mg GAE/100 g FW, 204.21 mg/100 g FW, and 66.24 mg/100 g FW, respectively, in comparison those of the plums at the immature and mid-ripe stages. The antioxidant capacity of the ripe plums was stronger than it was during the other stages of the plums growth. Among them, the total polyphenols of the ripe plums exhibited the strongest antioxidant capacity (IC_50_ values against DPPH and hydroxyl radicals were 28.19 ± 0.67 μg/mL and 198.16 ± 7.55 μg/mL, respectively), which was between the antioxidant capacity of the free polyphenols and bound polyphenols. The major phenolic monomer compounds of plum polyphenols were flavan-3-ols (epicatechin, catechin, proanthocyanidin, and procyanidin B_2_), flavonols (myricetin), and phenolic acids (chlorogenic acid, ferulic acid, and protocatechuic acid). Additionally, plum polyphenols exhibited a strong inhibitory effect on XOD, with an IC_50_ value of 77.64 μg/mL. The inhibition kinetics showed that plum polyphenols are mixed-type inhibitors that inhibit XOD activity and that the inhibition process is reversible. The calculated values of *K_i_* and *α* were 16.53 mmol/L and 0.26, respectively.

## 1. Introduction

Furong plums (*Prunus salicina* Lindl. cv. “furong”) are a fruit of the family Rosaceae and is a red-fleshed fruit characteristic of Fujian, China; they have a water content exceeding 85% and are more than 90 percent edible [1]. It is worth noting that Furong plums have a high content of polyphenol compounds, suggesting that they may have a strong antioxidant potential [2,3]. Furong plums can serve as a source of dietary polyphenols and have broad prospects for application [4]. The current research is focused on the effect of varieties on polyphenol content and biological activity, such as those of different plum varieties, including *Carissa macrocarpa*, *Zarechnaya rannyaya*, and *Ximenia caffra* [5,6,7,8]. Relatively less research has been conducted on the effect of maturity stages on the composition and activity of polyphenol compounds in plums. The research has shown that the antioxidant capacity of plums from South Africa, such as ruby red and PR04-19 plums, increases as they ripen [9]. In *Syzygium cumini*, the content of myricetin-3-O-hexoside increases with increasing maturity, while myricetin-3-O-pentoside and myricetin-3-O-rhamnoside show the opposite trend [10]. However, there are currently no research reports available on the composition of polyphenol compounds and antioxidant capacity at the different maturity stages of Furong plums.

Xanthine oxidase (XOD) is a key enzyme in the body responsible for the production of UA. It catalyzes the direct metabolic conversion of xanthine into UA or the conversion of hypoxanthine into xanthine, which is then further catalyzed to produce UA [11,12]. Hyperuricemia (HUA) is diagnosed when the uric acid (UA) levels exceed 420 μmol/L for males and 360 μmol/L for females [13,14]. HUA can cause acute or chronic gout, acute or chronic nephropathy, hypertension, coronary heart disease, and other diseases [15,16]. Furong plums are rich in bioactive polyphenols. Extensive research has found that natural polyphenol compounds can reduce the level of UA by inhibiting XOD activity and through other pathways, thus demonstrating a strong potential for lowering the level of uric acid. The examples include *Camellia japonica* bee pollen polyphenols, purple potato leaf polyphenols, and green tea polyphenols [17,18,19]. Furthermore, natural polyphenols from different plants will reduce the activity of XOD through different inhibition mechanisms. The inhibition mechanism of natural polyphenols on XOD can be analyzed through inhibition kinetics, and the inhibition types may be uncompetitive inhibition, competitive inhibition, and mixed-type inhibition [20,21]. However, there have been no research reports on the inhibition effect and mechanism of action of Furong plum polyphenols on XOD, indicating an urgent need for further study.

This experiment analyzed the content of polyphenols, total flavonoids, and anthocyanin as well as the antioxidant capacity of Furong plums at three different maturity stages to determine the most suitable maturity stage and polyphenolic compounds in Furong plums. Subsequently, the major phenolic monomer compounds of the polyphenols were analyzed, along with their inhibitory effect on the XOD enzyme activity and inhibition kinetics. The research on the polyphenols in Furong plums provides a theoretical basis for the medicinal value and food development of Furong plums.

## 2. Materials and Methods

### 2.1. Experimental Materials

**Figure 1:**
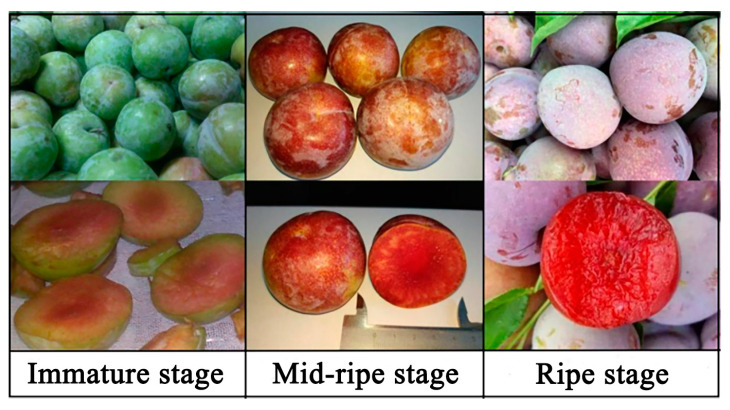
Images of harvested Furong plums at three different stages of fruit development.

Furong plums were collected from Yongtai, Fujian Province. To ensure similar growth conditions, the plums were all harvested from the same area (a 5 m × 5 m planting area) in the same orchard. The trees began bearing fruit on 1 March 2018, and the plums at three different maturity stages were harvested from 1 July 2018 to 10 August 2018. The experiment employed a random harvesting method, with three trees being harvested for each replicate. The collected plums were transported to a laboratory and refrigerated within 4 h. Figure 1 shows pictures of the plums harvested at three different maturity stages.

Immature stage: plums picked on 1 July 2018 (122 days after the fruit trees began to bear fruit), with lime green skin, about 30% red flesh, a hard texture, and 84.92 ± 1.05% water content.

Mid-ripe stage: plums picked on 19 July 2018 (140 days after the fruit trees began to bear fruit), with red skin, mostly red flesh, a hard texture, and 85.26 ± 1.86% water content.

Ripe stage: plums picked on 10 August 2018 (162 days after the fruit trees began to bear fruit), with dark red skin, equally dark red flesh, a soft texture, and 85.66 ± 1.75% water content.

Test sample preparation and storage: After the plum fruits were transported to the laboratory, selected fruits of uniform size, free from mechanical injuries and pests and diseases, were washed with distilled water to remove surface impurities. The sample surfaces were gently wiped clean with clean gauze, cut open, and de-nucleated; 250 g samples were taken according to the sampling requirements. The diagonal partitioning method was used, the diagonal part was taken, the tissue was quickly crushed, and then the samples were studied using the quartering method and packed into polyethylene plastic bottles. The processed fruits were rapidly frozen using liquid nitrogen, and then quickly transferred to an ultra-low temperature (−80 °C) environment.

### 2.2. Main Reagents

Phenol, sulfuric acid, gallic acid, forinol, anhydrous ethanol, ethyl acetate, n-hexane, potassium chloride, sodium acetate, hydrochloric acid, and acetone were analytically pure; methanol, acetonitrile, and formic acid were chromatographically pure. All of them were from Sinopharm Chemical Reagent Co., Ltd. (Shanghai, China); xanthine oxidase, xanthine (purity ≥ 98%), and allopurinol (purity ≥ 98%) were all from Yuanye Biotechnology Co., Ltd. (Shanghai, China).

### 2.3. Experimental Methods

#### 2.3.1. Extraction of Free Polyphenols

The extraction method was conducted following the literature described by Adom et al. [22], with certain modifications. A total of 20 g of Furong plum fruit samples were added to a 100 mL centrifuge tube, 50 mL of 80% chilled ethanol solution was added, it was homogenized for 3 min in a high-speed homogenizer (two times with an interval of 0.5 min), and then the supernatant was extracted three times via centrifugation (GL10MD, Xiangyi Group, Changsha, China) at 3500 r/min. Next, it was filtered and evaporated with a rotary evaporator (RE-301, Yuyao Xinbo Instrument Co., Ltd., Yuyao, China) at 45 °C under reduced pressure, and then fixed to 25 mL with ultrapure water, passed through a 0.45 μm organic-phase microporous membrane, and stored in a refrigerator at −40 °C.

#### 2.3.2. Extraction of Bound Polyphenols

The residue remaining after the extraction of free phenolics was collected; 20 mL of 2 mol/L NaOH solution was added and digested via stirring for 1.5 h, acidified with 6 mol/L HCl solution to pH 2, the fat of samples were removed by adding hexane and centrifugation, and the supernatant was collected three times by adding ethyl acetate and centrifuging. The filtrate was combined and evaporated using a rotary evaporator at 45 °C under reduced pressure, and then fixed with ultrapure water to 10 mL. The samples were subsequently filtered and stored in a refrigerator at −40 °C.

#### 2.3.3. Determination of Polyphenol Content

The experimental method was based on the procedure reported by Li et al. [23], with slight modifications. The polyphenol content was determined using the Folin–Ciocalteu colorimetric method. For the determination of the polyphenol content of plums, 200 μL of the plum polyphenol sample was taken and mixed with 2.5 mL of a 10% (*v*/*v*) forlinol solution. The polyphenol samples were shaken well and protected from light. Next, 2 mL of Na_2_CO_3_ solution with a mass of 75 g/L was added and mixed thoroughly. The mixture was allowed to stand in a water bath at 50 °C for 15 min after being made up to a specified volume. The absorbance of the samples were measured at 765 nm. Equation (1) represents the standard curve.

(1) $$y=0.6185x+0.1679\;(R^{2}=0.9909)$$

#### 2.3.4. Determination of Flavonoid Content

The different flavonoid contents were determined from free polyphenols, bound polyphenols, and total polyphenols, according to the method described by Hassanpour et al. [24] with some modifications. A total of 0.25 mL of the plum polyphenol sample and 0.3 mL of 10% Al(NO_3_)_3_ solution were added to 0.3 mL of 5% mass concentration of NaNO_2_ solution in a glass test tube. After allowing the mixture to stand, 4 mL of 1 mol/L NaOH solution was immediately added, supplemented with 65% ethanol to 10 mL, shaken, and mixed. The values of the samples were recorded at a wavelength of 510 nm. Equation (2) represents the standard curve.

(2) $$y=0.002x+0.0492\;(R^{2}=0.9995)$$

#### 2.3.5. Determination of Anthocyanin Content

According to the method of Zia et al. [25], a 10 g sample of plum fruit was added to 100 mL of 60% ethanol (acidified with hydrochloric acid, pH 3.0) extraction solution in a 250 mL container and extracted via 200 W ultrasonic-assisted extraction for 30 min in a 40 °C water bath under light avoidance. The supernatant of the samples was collected via centrifugation. The absorbance values were measured at wavelengths of 520 nm and 700 nm. The anthocyanin content of the extracts was calculated with Equations (3) and (4) using cyaniding-3-glucoside as the equivalent amount.

(3) $$\mathrm{Anthocyanins}\;(\mathrm{mg}/L)=\frac{\Delta A}{\varepsilon L}\times 10^{3}\times M\times n$$

(4) $$\Delta A=(A_{1}-A_{2})-(A_{3}-A_{4})$$

*A*_1_ and *A*_2_: absorbance at 520 nm and 720 nm (pH 1.0 buffer), respectively.

*A*_3_ and *A*_4_: absorbance at 520 nm and 720 nm (pH 4.5 buffer), respectively.

*L*: cuvette optical path length.

*ε*: molar absorbance coefficient of centaureidin-3-glucoside, 26,900 L/(mol·cm).

*M*: the molar mass of cyaniding-3-glucoside, 449.2 g/mol.

*n*: Dilution multiple. The final results were converted to the amount of anthocyanin contained in each mL.

#### 2.3.6. Determination of DPPH-Clearance

The solution of plum polyphenols was mixed with 0.1 mmol/L solution of DPPH. It was kept in the dark and allowed to stand for 30 min, followed by measurement at 517 nm. Equation (5) represents the formula for calculation.

(5) $$\mathrm{Clearance}\;\mathrm{rate}\;(\%)=\frac{A_{Blank}-A_{Sample}}{A_{Blank}}\times \;100\%$$

#### 2.3.7. Determination of Hydroxyl Radical Scavenging Rate

In a 10 mL screw-top test tube, a mixture of pure water, pH 7.4 phosphate buffer, fenugreek solution, EDTA-Na_2_Fe^2+^, and different concentrations of plum polyphenols were added. H_2_O_2_ was added to the samples at 40 °C and allowed to stand for 30 min. The absorbance was measured at 520 nm. Equation (5) represents the formula for calculation.

(6) $$\mathrm{Clearance}\;\mathrm{rate}\;(\%)=\frac{A_{Sample}-A_{Blank}}{A_{Contrast}-A_{Blank}}\times 100\%$$

#### 2.3.8. Identification of Phenolic Substance Components

The method of Jaiswal et al. [26] was used with slight modifications. For composition identification, 1 mL of the plum total polyphenol sample was taken, filtered through a 0.45 μm organic membrane, and analyzed using an HPLC (e2695, Waters Co., Ltd., Milford, MA, USA) instrument with a Sunfire C_18_ Chromatographic column (150 mm × 4.6 mm, 3.5 μm). The mobile phase consisted of 0.1% formic acid solution and 100% acetonitrile solution, with a flow rate of 0.7 mL/min. The Supplementary Materials reflects the experimental parameters of HPLC (Table S1).

### 2.4. Inhibition Mechanism of XOD

#### 2.4.1. XOD Activity Assay

The Furong plum polyphenols were dissolved in DMSO at different ratios, while XOD and xanthine were dissolved in PBS buffer. The XOD and xanthine solutions were reacted with polyphenol solutions of varying concentrations at room temperature, and the inhibitory capacity against XOD was determined by the absorbance at 290 nm. Allopurinol was the control sample. The inhibition rate of the sample on XOD can be calculated using the following Formula (7).

(7) $$\mathrm{Inhibition}\;\mathrm{rate}\;(\%)=\frac{V_{Enzyme}-V_{Sample}}{V_{Enzyme}-V_{Blank}}\times 100\%$$

#### 2.4.2. Inhibitory Kinetic Analysis of XOD

The experiment employed a Lineweaver–Burk plot to analyze the inhibition kinetics of Furong plum polyphenols on XOD, and it is described using the following Equations (8)~(10):

(8) $$\frac{1}{v}=\frac{K_{m}}{V_{\max}}\left(1+\frac{\left[I\right]}{K_{i}}\right)\frac{1}{\left[S\right]}+\frac{1}{V_{\max}}\left(1+\frac{\left[I\right]}{\alpha K_{i}}\right)$$

(9) $$\mathrm{Slope}=\frac{K_{m}}{V_{\max}}+\frac{K_{m}[I]}{V_{\max}K_{i}}$$

(10) $$\mathrm{Intercept}=\frac{1}{V_{\max}}+\frac{1}{\alpha K_{i}V_{\max}}[I]$$

In Equations (8)~(10), *V*: the rate of the enzymatic reaction; [*S*]: substrate concentration; [*I*]: inhibitor concentration; *K_m_*: Michaelis–Menten constant; *V*_max_: maximum reaction rate; *K_i_*: inhibition constant; *α*: apparent coefficient.

### 2.5. Statistical Analysis

Statistical data were collected using Origin 2019 software, and the data were subjected to one-way ANOVA using SPSS 21 software and significance analysis using Duncan’s multiple comparisons. Three groups of samples were measured, and the average was taken.

## 3. Results and Discussion

### 3.1. Polyphenol Content

In terms of the different forms of polyphenols, the free polyphenols account for over 90% of the total polyphenols in plums, while the bound polyphenols account for 7.83~8.72% of the total polyphenols. The bound polyphenols in plums are formed by a combination of polyphenols with cell wall components, such as cellulose, sugars, and proteins. These bound polyphenols mainly exist in the form of β-glycosides, where they are not subject to digestion by human digestive enzymes. They can only be absorbed after reaching the colon and have the potential for use in the treatment of colon cancer [27]. Other researchers have found that the bound phenolics in bananas exhibit anti-proliferative activity against colon cancer, with the activity of colon cancer cells decreasing as the concentration of bound phenolics increases [28].

**Figure 2:**
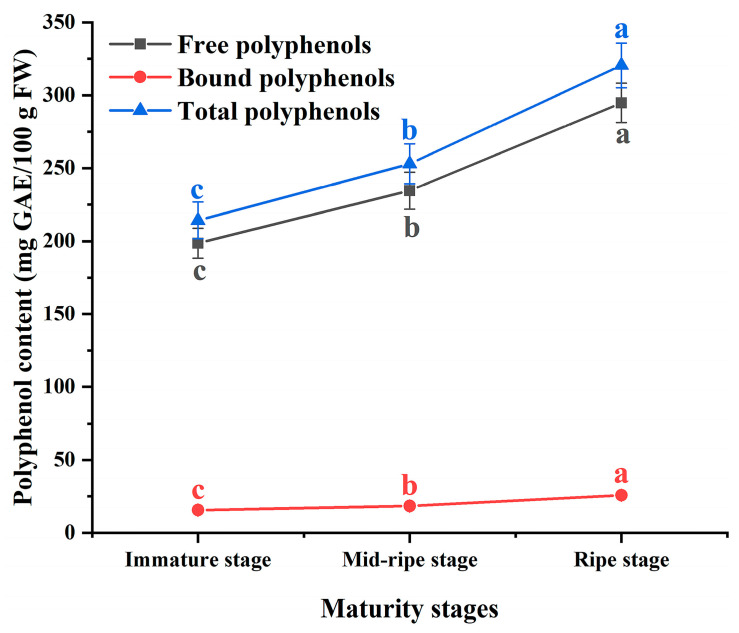
The effect of different maturity stages on the polyphenol content. The different letters indicate significant differences in the same group of polyphenols at different maturity stages (*p* < 0.05).

According to Figure 2, the total polyphenol content of the immature plums was 214.25 mg GAE/100 g FW (fresh weight), with the lowest content of bound polyphenols being only 15.69 mg GAE/100 g FW. The polyphenol content of the plums showed a rapid increase with maturity, reaching its highest value in the ripe plums; the total polyphenol content was 320.46 mg GAE/100 g FW, and the bound polyphenol content was 25.69 mg GAE/100 g FW. According to the previous studies, it was demonstrated that during the maturation stages of plums, there were significant changes observed in their polyphenol content. For instance, the polyphenol content in plum peel exhibited an increasing trend at different maturity stages [29], which is similar to the variation observed in the polyphenol content of the plums.

### 3.2. Flavonoid Content

**Figure 3:**
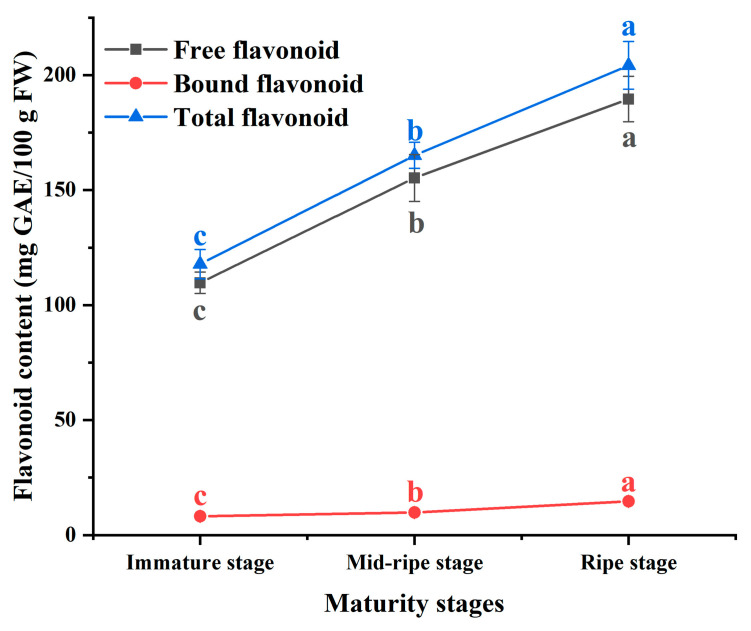
The effect of different maturity stages on the flavonoid content. The different letters indicate significant differences in flavonoid content at different maturity stages (*p* < 0.05).

Flavonoids are secondary metabolites and possess a polyphenolic structure. Extensive research has demonstrated that flavonoid compounds extracted from natural plants possess potent antioxidant capabilities and exhibit excellent inhibitory effects on XOD [30,31]. Figure 3 showed the trends in flavonoid content at different maturity stages. The total flavonoid content of the plums ranged from 117.84 to 204.21 mg GAE/100 g FW. The flavonoid content increased with increasing maturity (*p* < 0.05). At different maturity stages, the free flavonoid content was significantly higher than the bound flavonoid content (*p* < 0.05), constituting over 90% of the total flavonoid content.

### 3.3. Anthocyanin Content

**Figure 4:**
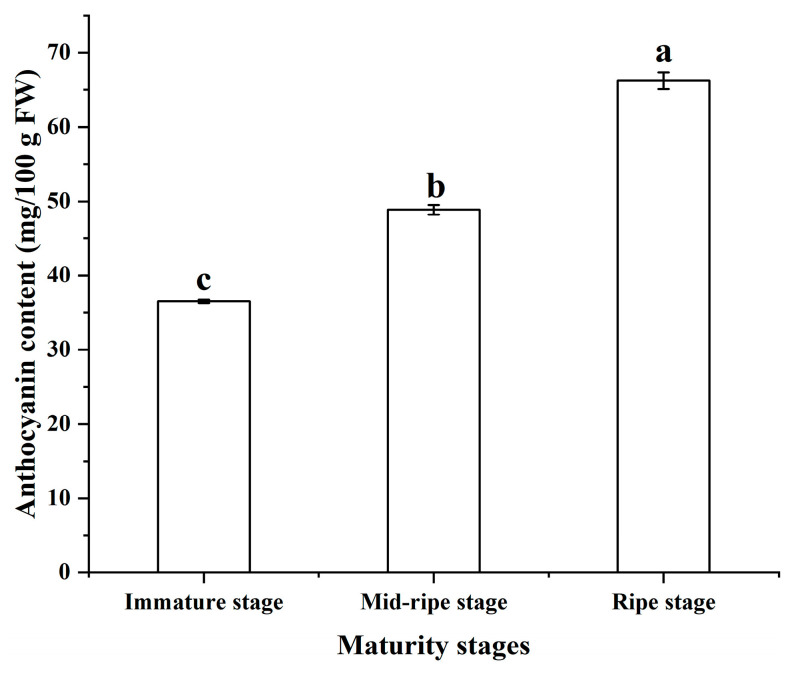
The effect of different maturity stages on the anthocyanin content. The different letters indicate significant differences in anthocyanin content at different maturity stages (*p* < 0.05).

Anthocyanins are unique natural polyphenols and water-soluble pigments that possess excellent anti-inflammatory, anticancer, and antioxidant properties [32]. They are one of the main pigments found in dark-colored plants and plant parts, such as petals, berries, and vegetables [33]. As shown in Figure 4, the anthocyanin content of plums gradually increases as they ripen, reaching its highest level at full ripeness, with a content of 66.24 mg/100 g FW. The accumulation of anthocyanins caused the change in color of the plums, which was also the reason why the plums gradually turn red during different maturity stages [34].

### 3.4. Antioxidant Capacity

The scavenging ability of the polyphenols on DPPH were shown in Table 1. With the increase in ripeness, the DPPH scavenging ability of plum polyphenols was gradually enhanced, and the IC_50_ (half-maximal inhibitory concentration) of the free, bound, and total polyphenols for DPPH scavenging was the highest for both the ripe plums, 38.57 μg/mL, 22.69 μg/mL and 28.19 μg/mL, respectively. It can be seen that with the ripening of the fruits, the increase in polyphenols made the fruits have a higher antioxidant activity level. This indicated that with an increase in polyphenol concentration, the DPPH radical scavenging ability also increased [35].

Hydroxyl radical (·OH) in the body can cause oxidative damage, reducing the cellular activity, and leading to cell injury and death, thereby affecting the normal physiological functions of the organism [36]. According to Table 2, the scavenging level of the bound polyphenols on hydroxyl radicals was about twice as high as that of the free polyphenols; this could be because the bound polyphenols were obtained using the base hydrolysis extraction method, and other research has shown that a higher polyphenol content can be obtained using the base hydrolysis method, thus resulting in stronger antioxidant capabilities [37]. The total polyphenols exhibited excellent antioxidant activity, which was found to be stronger than that of the free polyphenols. The IC_50_ values of the total polyphenols had a range of 188.57~198.16 μg/mL. Additionally, the ripeness of the plums did not affect the scavenging ability of the hydroxyl radicals.

Compared with the immature plums and mid-ripe plums, the antioxidant capacity of the ripe plums was the strongest. Among them, the total polyphenols in the ripe plums exhibited a stronger antioxidant capacity (with IC_50_ values of 28.19 ± 0.67 μg/mL for DPPH and 198.16 ± 7.55 μg/mL for the hydroxyl radicals), which was between the antioxidant capacity of the free and bound polyphenols of the ripe plums. Bound polyphenols are polyphenol compounds that are closely linked to the food matrix, and their low extraction rate limits their application in food and subsequent bioaccessibility [38,39]. On the other hand, the extraction rate of total polyphenols was higher than that of the free and bound polyphenols, and they exhibited excellent antioxidant capabilities. In short, plum polyphenols demonstrate a strong overall antioxidant capacity, and the reason for their potent antioxidant ability can be further elucidated by analyzing the composition of the plum’s phenolic monomer compounds. Taking into consideration both the extraction rate and antioxidant activity, we selected the total polyphenols extract from the ripe plums for studying the major phenolic monomer compounds and their inhibition mechanism on XOD.

### 3.5. Major Phenolic Monomer Compounds

**Figure 5:**
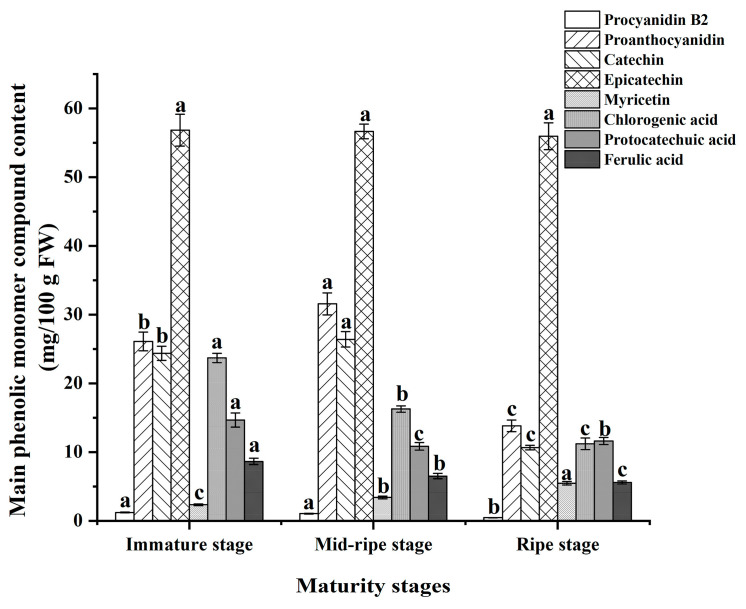
Contents of phenolic monomers in Furong plums at different maturity stages. Note: different letters indicate significant differences in compounds at different maturity stages (*p* < 0.05).

As shown in Figure 5, HLPC detected nine phenolic substances in plums, primarily in the form of flavan-3-ols (epicatechin, catechin, proanthocyanidin, and procyanidin B_2_), and phenolic acids (chlorogenic acid, ferulic acid, and protocatechuic acid). Additionally, flavonols (myricetin) were also present.

Epicatechin was the most abundant phenolic substance in the plums, with a content of 55.96~56.85 mg/100 g FW, and ripeness had no significant effect on the epicatechin content, indicating that Furong plums can maintain a higher level of catechin content during the ripening process. Catechin and epicatechin are isomers of each other, and both belong to catechins. As the ripeness increased, the content of catechin in plums decreased, and the lowest catechin content was 10.68 mg/100 g FW in the ripe plums.

The proanthocyanidin and procyanidin B_2_ contents of the plums were 13.83~26.11 mg/100 g FW and 0.48~1.24 mg/100 g FW, respectively. The content of proanthocyanidin in the ripe plums significantly decreased (*p* < 0.05). The research indicated that the immature fruits contained higher levels of proanthocyanidins because proanthocyanidin had a bitter and astringent taste, which can protect immature fruits. As the fruits matured, there was a redirection of the flavonoid biosynthetic pathway from proanthocyanidin production to anthocyanin production. This resulted in a decrease in the content of proanthocyanidin and an increase in the anthocyanin content, leading to a noticeable change in the color of the plums [40,41]. This conclusion was consistent with that of measurements of the anthocyanin content (Section 3.3).

Myricetin is a flavonol compound, and the content of myricetin in the plums significantly increased with maturity (*p* < 0.05) to 2.35~5.49 mg/100 g FW. The trend was the same as the trend of increasing flavonoid content in the plums.

The chlorogenic acid, protocatechuic acid, and ferulic acid contents in the plums decreased significantly with the increase in maturity (*p* < 0.05). The contents of chlorogenic acid, protocatechuic acid, and ferulic acid in the plums were 11.23~23.71 mg/100 g FW, 10.87~14.67 mg/100 g FW, and 5.59~8.66 mg/100 g FW, respectively. This research has indicated that the decrease in phenolic compounds is associated with a reduction in primary metabolism in ripe fruits, leading to a scarcity of substrates required for the biosynthesis of phenolic compounds. Thereby, ripe fruits have low levels of phenolic acids [42].

The composition, content, and structure of phenolic monomer compounds can affect the antioxidant capacity of plum polyphenols. In our previous antioxidant experiment, we discovered the strong antioxidant capability of plum polyphenols. The strong antioxidant ability of flavan-3-ol is achieved through hydroxyl substitution, where the number of OH groups and their positions on the phenyl ring are crucial factors. The strong antioxidant capability of plum polyphenols is primarily due to the high content of epicatechins, which is achieved through the ortho-dihydroxy group substitution in the B-ring [43]. Proanthocyanidin is also the main component of plum phenolic compounds, possessing a strong antioxidant capability. Interestingly, the content of proanthocyanidin significantly decreased during the ripening process. However, the antioxidant ability of plum polyphenols showed an upward trend. This could be attributed to the increase in anthocyanin content, which replaced proanthocyanidin in the antioxidant process [44,45]. The content of myricetin significantly increased during the ripening process. It has been reported that myricetin exhibits a stronger antioxidant capability than epicatechins. This is attributed to myricetin’s ability to chelate transition metal ions, which is far superior to that of epicatechins. This indicates that although myricetin has a lower content, it still contributes significantly to the antioxidant capability of plum polyphenols [46,47]. In short, the Furong plum polyphenols represent a complex mixture, and the dose–response relationship and structure–activity relationship between its different phenolic compounds and their antioxidant capacities are highly intricate.

### 3.6. The Effect of Polyphenols from Furong Plums on the Inhibition Rate of XOD

**Figure 6:**
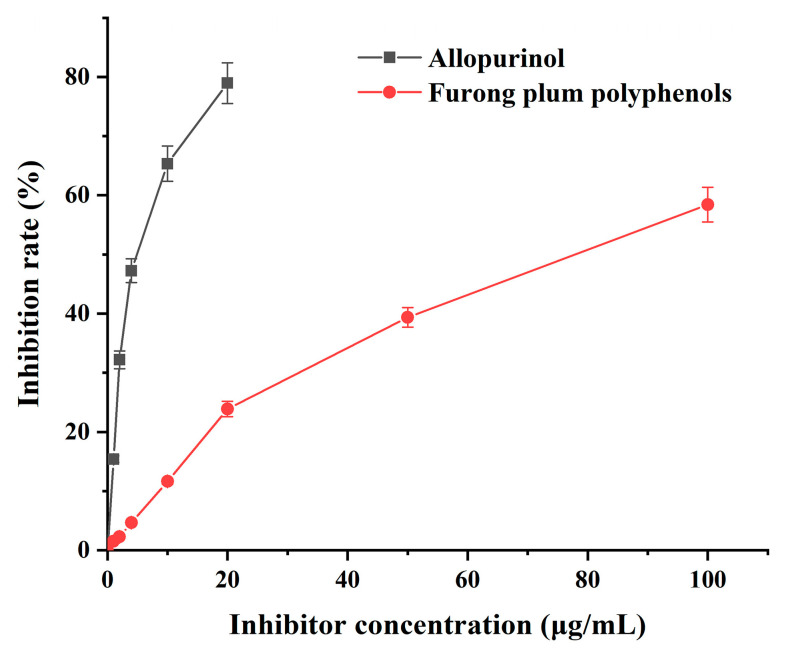
Inhibition rate of Furong plum polyphenols on XOD.

According to Figure 6, as the mass concentration of plum polyphenols increased, the inhibition rate of XOD continuously increased. The polyphenols exhibited a certain concentration-dependent relationship with their inhibition of XOD. The IC_50_ values for the plum polyphenols and the positive control (allopurinol) were 77.64 μg/mL and 4.95 μg/mL, respectively, indicating that Furong plum polyphenols had a certain inhibitory effect on XOD. According to the literature, Mo-pterin is reported to be the critical active site for inhibiting XOD, and its interaction with polyphenol compounds is specific [48]. Moreover, this may be attributed to the fact that Furong plum polyphenols are primarily composed of monomeric phenolic compounds, such as catechin and epicatechin. The hydroxyl groups at positions three, five, and seven of the chroman ring in catechin and epicatechin readily form hydrogen bonds to inhibit XOD activity [49].

### 3.7. Reversibility Analysis of XOD Inhibition by Furong Plum Polyphenols

**Figure 7:**
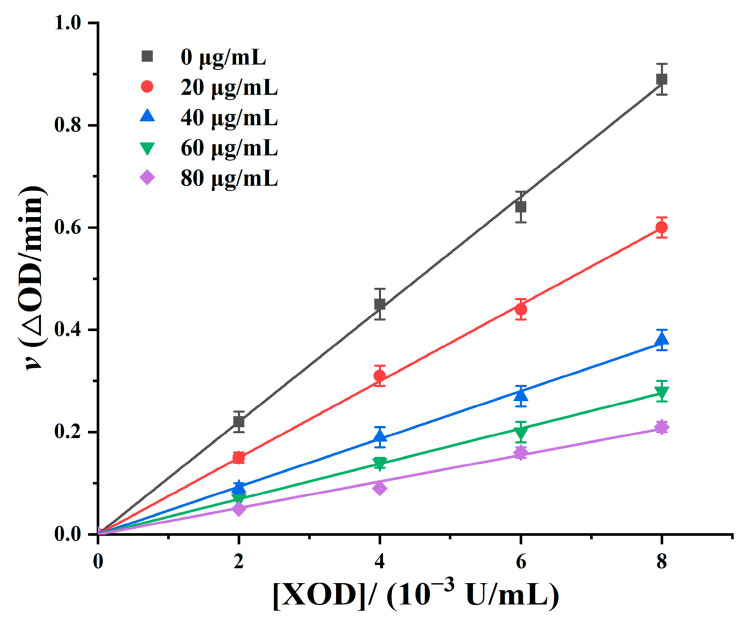
Plots of ν vs. XOD. c(xanthine) = 4.0 × 10^−4^ mol/L, and c(Furong plum polyphenols) = 0, 20, 40, 60, and 80 μg/mL.

Figure 7 shows a relationship graph between the reaction rate and XOD concentration under the influence of different concentration of polyphenol inhibitors. Each line passes through the origin; while the polyphenol content increased, the slope gradually decreased. This indicates that the inhibition of XOD by plum polyphenols is a reversible process [50]. This may be attributed to the abundance of -OH functional groups present in the structure of polyphenols from the plums, which reversibly bound to the essential groups of XOD through non-covalent bonds, resulting in the inhibition of enzyme activity, rather than by reducing the effective amount of XOD to inhibit enzyme activity [51].

### 3.8. Inhibitory Mechanism of Furong Plum Polyphenols on XOD

**Figure 8:**
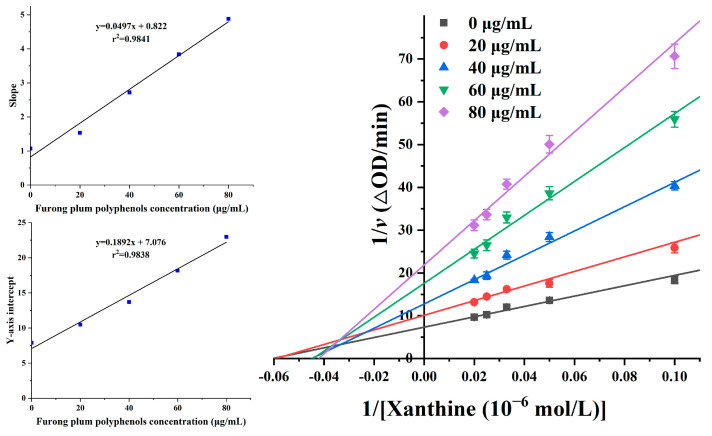
Lineweaver–Burk plots. c(XOD) = 5.0 × 10^−3^ U/mL, c(Furong plum polyphenols) = 0, 20, 40, 60, and 80 μg/mL. Insert: the secondary plot of slope and Y intercept against the concentration of Furong plum polyphenols.

There are many XOD inhibitors extracted from plants, but the different extracts exhibit different types of XOD inhibition. For example, in the total flavonoid extract from *Ginkgo biloba* leaves, kaempferol is a competitive XOD inhibitor, while the other flavonoid compounds were anticompetitive inhibitors. The water extract from *Perilla frutescens* leaves exhibited mixed inhibition properties [52,53]. Plum polyphenols have a significant inhibitory effect on XOD. The inhibition kinetics of plum polyphenols were studied using a Lineweaver–Burk double reciprocal plot (Figure 8) to analyze the changes in *K_m_* and *V*_max_. The slope represents the ratio of *K_m_* to *V*_max_, while the y intercept represents the value of 1/*V*_max_.

From Figure 8, it can be seen that all the lines with different slopes and intercepts corresponding to different concentrations of plum polyphenols intersecting in the second quadrant. However, the slopes and intercepts of all the lines are not the same. Moreover, as the concentration of the plum polyphenols inhibitor increased, both the slope and *Y* axis intercept increased. This indicated that *K_m_* gradually increased and V_max_ gradually decreased. Based on this observation, we inferred that plum polyphenols acted as a mixed-type inhibitor to inhibit XOD. This suggested that plum polyphenols could compete with xanthine at the active site of XOD and also bind to other sites of XOD to inhibit the formation of the enzyme–substrate complex [54].

In addition, the concentration of plum polyphenols are linearly fitted with the slopes and intercepts in a secondary replot, showing a good linear relationship, with r^2^ values of 0.9841 and 0.9838, respectively. This indicated that the inhibitory effect of Furong plum polyphenols on XOD was accomplished through a single inhibition site or a single class of inhibition site. The calculated values of *K_i_* and *α* were 16.53 mmol/L and 0.26, respectively.

## 4. Conclusions

This experiment was concluded at different maturity stages (immature stage, mid-ripe stage, and ripe stage) and with different polyphenol compositions (total polyphenols, free polyphenols and bound polyphenols) of Furong plums to determine the most suitable processing parameters and to study the major phenolic monomer compounds of Furong plum polyphenols as well as their inhibition mechanism on XOD. The experimental results indicated that the total polyphenols of plums at the ripe stage were the most suitable object for study, as they had the highest polyphenol, flavonoid, and anthocyanin contents and demonstrated an excellent antioxidant capacity. The composition of total polyphenols of the plums primarily consisted of flavan-3-ols (epicatechin, catechin, proanthocyanidin, and procyanidin B_2_), flavonols (myricetin), and phenolic acids (chlorogenic acid, ferulic acid, and protocatechuic acid). Furthermore, the plum polyphenols acted as a mixed-type inhibitor in the XOD inhibition kinetics experiment, and the inhibition process was a reversible process, with only one inhibitory binding site. In conclusion, the total polyphenols of Furong plum at the ripe stage exhibit an excellent research value, but further investigation was needed to understand their antioxidant effect and potential mechanism in vivo.

## Figures and Tables

**Table 1:** IC_50_ for scavenging DPPH radicals by Furong plum polyphenols at three maturity stages.

Maturity Stages	IC_50_ of the DPPH Scavenging (μg/mL)		
	Free Polyphenols	Bound Polyphenols	Total Polyphenols
Immature stage	98.48 ± 1.57 a	39.14 ± 1.02 a	59.63 ± 1.28 a
Mid-ripe stage	45.99 ± 1.02 b	29.67 ± 0.48 b	34.28 ± 0.54 b
Ripe stage	38.57 ± 0.84 c	22.69 ± 0.58 c	28.19 ± 0.67 c

**Note:** Different letters indicate significant differences in IC_50_ of the DPPH scavenging with different maturity stages (*p* < 0.05).

**Table 2:** IC_50_ for ·OH scavenging by Furong plum polyphenols with three maturity stages.

Maturity Stages	IC_50_ of ·OH Scavenging (μg/mL)		
	Free Polyphenols	Bound Polyphenols	Total Polyphenols
Immature stage	249.63 ± 7.40 a	121.42 ± 6.92 a	192.69 ± 5.48 a
Mid-ripe stage	246.81 ± 7.42 a	126.65 ± 3.68 a	188.57 ± 6.81 a
Ripe stage	253.66 ± 8.96 a	116.78 ± 4.54 a	198.16 ± 7.55 a

**Note:** Different letters indicate significant differences in IC_50_ of hydroxyl radical scavenging with different maturity stages (*p* < 0.05).

## Data Availability

The data are contained within the article or Supplementary Material.

## Supplementary Materials

The following supporting information can be downloaded at: https://www.mdpi.com/article/10.3390/foods12234253/s1, Table S1. Gradient elution procedures.
