# Supplementary material for: Polyphenol Composition, Antioxidant Capacity and Xanthine Oxidase Inhibition Mechanism of Furong Plum Fruits at Different Maturity Stages
Source: Foods. 2023 Nov 24;12(23):4253. doi: 10.3390/foods12234253 (PMC10705914; doi:10.3390/foods12234253)
Supplement: Supplementary file 1 [file foods-12-04253-s001.zip › foods-2712272-SI.pdf]

1. The experimental parameters of HPLC

Chromatographic column: Sunfire C_{18} 150 mm × 4.6 mm 3.5 μm

Analytical standard: proanthocyanidin (chromatographic purity), procyanidin B2 (chromatographic purity), catechin (chromatographic purity), epicatechin (chromatographic purity), myricetin (chromatographic purity), chlorogenic acid (purity ≥ 98%), protocatechuic acid (purity ≥ 99%), ferulic acid (chemically pure), all for Shanghai Yuanye Biotechnology Co., Ltd., China.

Mobile phase A: 0.1% formic acid solution. Mobile phase B: 100% acetonitrile solution. Table S1 showed the Gradient elution procedures.

Table S1. Gradient elution procedures.

Time (min)	Mobile phase A (%)	Mobile phase B (%)
0-5	95	5
5-35	95-75	5-25
35-50	75-60	25-40
50-55	60	40
55-60	60-95	40-5
60-70	95	5

The flow rate of HPLC was 0.7 mL/min, the injection volume was 20 μL, the column temperature was 40℃, and the detection wavelength was 280 nm.
